# Supplementary material for: The sand fly (Diptera: Psychodidae) fauna of the urban area of Lassance, Northeast Minas Gerais, Brazil
Source: PLoS One. 2021 Oct 13;16(10):e0257043. doi: 10.1371/journal.pone.0257043 (PMC8513856; doi:10.1371/journal.pone.0257043)
Supplement: Attachment — Submitted filename: Response to editor.docx [file pone.0257043.s001.docx]

August 13, 2021

Manuscript **PONE-D-21-17111**

Dear Albert Schriefer, M.D., Ph.D.,

Thank you for submitting the article and for the reviewers' suggestions.

Below are our responses to Editor's and Reviewers' comments.

Bewick et al. [5] - **Our article did not cite this unpublished work**.

Reviewer #1 We consider the work suitable for publication. We can raise as a question the lack of statistical support to affirm the relationship between relative humidity and the abundance of collected sandflies. Additionally, we miss a discussion with the literature on the finding of natural infection of Sc. sordelli by Leishmania. Reference indication follows below:

Da-Silva YY, Sales KGS, Miranda DEO, Figueredo LA, Brandão-Filho SP, Dantas-Torres F. Detection of Leishmania DNA in Sand Flies (Diptera: Psychodidae) From a Cutaneous Leishmaniasis Outbreak Area in Northeastern Brazil. Journal of Medical Entomology, Volume 57, Issue 2, March 2020, Pages 529–533.

**The reference requested in a paragraph on the detection of *Leishmania* in *Sciopemyia* *sordellii* has been included (pages 272-275).**

**Climatic data were presented in general. The information generated was collected in another municipality (through INMET - Brazilian meteorological agency), which could cause incorrect information. In our next projects, we will carry out these measurements in loco, with devices (thermometers, hygrometers and pluviometer) individualized for each trap.**

Reviewer #2: The objective of the study is relevant considering the expansion of visceral leishmaniasis in urban areas.

Abstract:

Line 40: Include the meaning of NMDS. **It was included in full.**

Methods:

Lines 84 and 91 start with the same words, I suggest a slight change. **The sentence has been changed.**

Line 113: Sacrificed is not a good term. **We removed the term "sacrificed".**

Best Regards

Sincerely,

José Dilermando Andrade Filho

Instituto René Rachou

Oswaldo Cruz Foundation
